# Annual Announcement of the Rush Medical Col. of Chicago, Ill.

**Published:** 1851-10

**Authors:** 


*Annual Announcement of the Rush Medical College of Chicago, Illinois. Session of* 1851–2.

We are pleased to perceive from the catalogue of students accompanying the above announcement, that the institution is in a flourishing condition. The lectures for the coming session, will commence the 3d of November, and continue sixteen weeks. Candidates for the degree of Doctor of Medicine, are required to be twenty-one years of age; to have pursued the study of medicine three years, and to have attended two courses of lectures, the last to be in this institution. He must also have taken the dissecting and hospital ticket during, at least, one college term.

